# Multimodal Musculoskeletal Rehabilitation in Clinical Practice: A Bibliometric and Altmetric Mapping Study (1989–2026)

**DOI:** 10.3390/healthcare14111564

**Published:** 2026-06-03

**Authors:** Nurmuhammet Taş

**Affiliations:** Clinic of Physical Medicine and Rehabilitation, Erzurum City Hospital, Erzurum 25070, Turkey; nu_mu_ta@hotmail.com; Tel.: +90-532-338-02-37

**Keywords:** multimodal rehabilitation, musculoskeletal rehabilitation, exercise therapy, self-management, biopsychosocial model, guideline implementation

## Abstract

**Background:** Multimodal rehabilitation represents standard practice in musculoskeletal care, where exercise therapy is routinely combined with manual therapy, electrotherapy, education, and cognitive–behavioral strategies. However, research has largely evaluated these modalities in isolation, and no bibliometric synthesis has characterized multimodal rehabilitation despite its predominance in routine practice. **Objective:** To characterize global research activity, thematic clusters, and diagnostic patterns underpinning multimodal musculoskeletal rehabilitation and to examine their alignment with contemporary rehabilitation guidelines and practice models. **Methods:** A bibliometric and altmetric analysis was performed using Web of Science Core Collection (1989–2026). Studies indexed under exercise therapy, manual therapy, electrotherapy, education, and cognitive–behavioral approaches were included. Network analyses (co-occurrence, co-authorship, thematic evolution, and bibliographic coupling) were conducted using Bibliometrix and VOSviewer. Diagnostic subgroups included osteoarthritis, low back pain, chronic musculoskeletal pain, tendinopathy, and shoulder disorders. **Results:** A total of 409 publications were identified. Five multimodal combinations were recurrent: exercise + education, exercise + cognitive–behavioral therapy, exercise + manual therapy, exercise + electrotherapy, and mixed multimodal programs. Diagnostic subgrouping showed distinct patterns, with osteoarthritis and low back pain clustering around exercise + education, chronic musculoskeletal pain around exercise + CBT/self-management, and tendinopathy/shoulder disorders around exercise + manual therapy. Temporal analyses demonstrated a shift from unimodal electrophysical agents toward guideline-aligned biopsychosocial models. Altmetric signals suggested relevant dissemination and policy attention. **Conclusions:** Multimodal musculoskeletal rehabilitation is research-intensive, diagnosis-specific, and aligned with guideline recommendations prioritizing exercise, education, self-management, and behavioral strategies. These findings support multimodal rehabilitation as a maturing evidence-based practice model with implications for pragmatic trials, guideline implementation, and clinical service delivery. Beyond research implications, these patterns are relevant for musculoskeletal care pathways, training of rehabilitation professionals and health system planning.

## 1. Introduction

Musculoskeletal disorders are among the leading contributors to disability worldwide, imposing substantial personal, social, and economic burden across the lifespan [1,2,3,4]. Conditions such as osteoarthritis, low back pain, chronic musculoskeletal pain, tendinopathies, and shoulder disorders frequently impair mobility, function, and participation and constitute a major driver of health care utilization [1,2,3,4,5,6]. In rehabilitation practice, the management of these disorders rarely relies on a single modality. Instead, multimodal interventions that combine exercise therapy, manual therapy, electrotherapy, patient education, and cognitive–behavioral approaches represent the prevailing pattern of care [7,8,9]. In addition, emerging integrative and mind–body rehabilitation approaches such as yoga-based rehabilitation programs have increasingly been investigated within musculoskeletal care. These interventions typically combine physical exercise, breathing regulation, body awareness, behavioral strategies, and patient-centered self-management components, thereby conceptually aligning with contemporary multimodal and biopsychosocial rehabilitation frameworks [10,11,12].

Across the past two decades, musculoskeletal rehabilitation has undergone a paradigm shift from unimodal biomedical interventions toward biopsychosocial, self-management, and guideline-based models of care [13,14,15,16]. International guidelines for osteoarthritis, low back pain, and chronic pain increasingly emphasize the integration of exercise therapy with education, behavioral strategies, and self-management rather than passive or isolated electrophysical agents [17,18,19,20]. For example, the American College of Physicians recommends exercise therapy and cognitive–behavioral interventions for low back pain [17]; the Osteoarthritis Research Society International identifies exercise and education as core recommendations for knee and hip osteoarthritis; and the International Association for the Study of Pain advocates for biopsychosocial and multimodal rehabilitation strategies in chronic pain [18]. Similar trends are reflected in national physiotherapy and rehabilitation guidelines, reinforcing the role of multimodal care pathways in real-world clinical practice [19,20,21,22].

Despite this evolution, the research literature has historically examined rehabilitation modalities in isolation. Exercise therapy, manual therapy, electrotherapy, and behavioral interventions have each been the focus of numerous randomized trials, systematic reviews, and bibliometric studies [23,24,25,26,27], yet the intersection of these modalities has rarely been quantified at scale. As a result, the scientific knowledge base remains fragmented relative to how rehabilitation is delivered in practice, limiting the ability to understand thematic evolution, clinical relevance, and evidence gaps across multimodal care.

To date, no bibliometric study has mapped multimodal physical therapy interventions in musculoskeletal rehabilitation. Existing bibliometric syntheses have focused on single modalities (e.g., exercise; manual therapy) or specific diagnostic entities (e.g., osteoarthritis; low back pain) [28,29,30,31], but none have characterized how exercise, manual therapy, electrotherapy, behavioral interventions, and education co-occur or cluster within rehabilitation research. Furthermore, little is known about how such combinations align with guideline priorities, biopsychosocial models, and self-management strategies or how they vary across diagnostic groups.

The present study addresses this gap by conducting a bibliometric and altmetric mapping of multimodal musculoskeletal rehabilitation. We sought to (i) identify thematic clusters of combined rehabilitation modalities; (ii) examine diagnostic subgroup patterns across osteoarthritis, low back pain, chronic musculoskeletal pain, tendinopathy, and shoulder disorders; (iii) characterize temporal and thematic evolution from unimodal to biopsychosocial rehabilitation; and (iv) explore dissemination and policy attention through digital altmetrics. By contextualizing multimodal rehabilitation within contemporary guidelines and practice models, this work aims to contribute to a more integrated understanding of rehabilitation science and its clinical application.

## 2. Materials and Methods

### 2.1. Data Collection and Search Strategy

On 10 January 2026, a comprehensive literature search was conducted using the Web of Science Core Collection to identify publications related to multimodal musculoskeletal rehabilitation. The search specifically included the Science Citation Index Expanded (SCI-EXPANDED; coverage: 1900–present) and the Social Sciences Citation Index (SSCI; coverage: 1956–present). The coverage characteristics of these sub-datasets were explicitly reported to improve methodological transparency and reproducibility in accordance with recommendations from previous scientometric studies emphasizing detailed reporting of Web of Science Core Collection data sources [32,33,34].

The Web of Science Core Collection was selected because of its standardized indexing structure, citation tracking reliability, compatibility with major bibliometric software tools (e.g., VOSviewer version 1.6.20 (Centre for Science and Technology Studies, Leiden University, Leiden, The Netherlands), CiteSpace version 6.3.R1 (Drexel University, Philadelphia, PA, USA), and Bibliometrix version 4.3.2 (University of Naples Federico II, Naples, Italy)), and widespread use in bibliometric and scientometric research [32,33,34]. In addition, Web of Science provides highly structured metadata and consistent citation indexing, which facilitate reproducible network visualization, co-citation analysis, and thematic mapping. Although databases such as Scopus may provide broader journal coverage in some disciplines, Web of Science Core Collection was considered more suitable for the present study because of its methodological consistency and established use in bibliometric mapping studies [32,33,34]. Nevertheless, previous scientometric studies have noted that the coverage and indexing policies of the Web of Science Core Collection have expanded over time, which may influence longitudinal bibliometric analyses and publication growth trends [33,34].

The search strategy was designed to preferentially identify studies integrating multiple rehabilitation modalities within the same therapeutic framework, including exercise therapy, manual therapy, electrotherapy, patient education, and cognitive–behavioral interventions. A total of 409 records were retrieved and included in the bibliometric and altmetric analyses. The search strategy and retrieval process are summarized in Table 1. In addition, several bibliometric visualization and clustering parameters (e.g., threshold selection and network density optimization) were adjusted to improve the interpretability of network visualizations, which may introduce a degree of methodological variability in thematic mapping results.

Unlike studies that apply multiple independent search sets followed by Boolean intersections, the present study employed a single comprehensive search query that simultaneously incorporated exercise, manual therapy, electrotherapy, education, and cognitive–behavioral rehabilitation-related terms. Retrieved records were subsequently screened for eligibility and thematic relevance at the title and abstract level using predefined inclusion and exclusion criteria focused on musculoskeletal rehabilitation relevance and multimodal intervention structure. Records lacking clear musculoskeletal relevance or sufficient multimodal rehabilitation content were excluded following consensus review.

Because the coverage and indexing policies of the Web of Science Core Collection have evolved over time, historical bibliometric trends should be interpreted with consideration of potential database-dependent variations in retrospective coverage, indexing practices, and metadata consistency, particularly for older literature from the late 1980s and early 1990s [34].

The search strategy was intentionally designed to prioritize conceptual specificity by identifying studies explicitly integrating multiple rehabilitation components within the same therapeutic framework. However, certain multidisciplinary, integrative, or mind–body rehabilitation approaches (e.g., yoga-based rehabilitation models) involving broader therapeutic frameworks, fewer combined modalities, or alternative terminology may not have been fully captured within the final dataset.

### 2.2. Screening and Eligibility

Records were included if they involved musculoskeletal conditions and at least two combined rehabilitation modalities from the search taxonomy. The search strategy was designed to prioritize studies explicitly integrating multiple rehabilitation components within the same therapeutic framework rather than isolated unimodal interventions. Exclusion criteria included (i) non-musculoskeletal conditions (e.g., stroke; cardiovascular disease), (ii) animal or basic science studies, and (iii) duplicates. Screening and eligibility assessment were performed manually at the title and abstract level using predefined thematic eligibility criteria focused on musculoskeletal rehabilitation relevance and multimodal intervention structure.

### 2.3. Variable Extraction

For each publication, the following metadata were extracted: year of publication, country, institution, journal, authors, keywords, citations, funding agency, and document type. Rehabilitation modalities were coded as exercise therapy, manual therapy, electrotherapy, education, and cognitive–behavioral approaches.

### 2.4. Classification of Intervention Modalities

To facilitate clinical interpretation of the bibliometric findings, the included publications were conceptually classified according to multimodal rehabilitation intervention domains combining exercise therapy with manual therapy, electrotherapy, education, and cognitive–behavioral strategies. This classification reflects the most frequently reported multimodal rehabilitation approaches identified in the literature and provides a clinical framework for contextualizing the bibliometric and altmetric results in relation to contemporary guideline recommendations (Table 2).

The resulting classification was used as an analytical schema for subsequent thematic, clustering and diagnostic subgroup analyses, enabling integration of bibliometric outputs with real-world rehabilitation practice patterns and care pathways.

### 2.5. Diagnostic Subgroup Coding

Publications were categorized into diagnostic subgroups: osteoarthritis, low back pain, chronic musculoskeletal pain, tendinopathy, and shoulder disorders. Diagnostic mapping was based on author keywords, title terms, and abstract terminology.

### 2.6. Bibliometric and Network Analysis

Descriptive bibliometric analyses were performed to summarize key characteristics of the multimodal rehabilitation literature, including annual publication output, source journals, citation patterns, authorship characteristics, and institutional and country contributions. Keyword frequencies were analyzed to identify commonly reported rehabilitation modalities and musculoskeletal diagnostic terms. Basic descriptive statistics and temporal trend analyses were conducted in Microsoft Excel 365 (Microsoft Corporation, Redmond, WA, USA). Similar long-term bibliometric approaches have been applied in musculoskeletal rehabilitation and related clinical fields to map influential contributors, research frontiers, and thematic evolution over time (Ozyigit et al., 2024) [35]. International collaboration was assessed using multiple-country publications (MCP) to characterize cross-national research partnerships, whereas single-country publications (SCP) were used to represent domestic research activity.

Bibliometric and visualization analyses were performed using VOSviewer version 1.6.20 (Centre for Science and Technology Studies, Leiden University, Leiden, The Netherlands), CiteSpace version 6.3.R1 (College of Computing and Informatics, Drexel University, Philadelphia, PA, USA), and Bibliometrix version 4.3.2 (Department of Economics and Statistics, University of Naples Federico II, Naples, Italy). VOSviewer was used for co-authorship, co-citation, and keyword co-occurrence analyses as well as visualization of collaboration networks among authors, institutions, and countries. Minimum occurrence thresholds were flexibly adjusted according to network density and visualization interpretability to identify influential authors, journals, and thematic clusters. For network visualization, node size reflected publication or citation frequency, whereas link strength represented collaboration intensity or co-occurrence relationships between items.

CiteSpace was used for thematic clustering, timeline visualization, and temporal mapping analyses using the log-likelihood ratio (LLR) clustering algorithm and time-slicing procedures. Cluster views and timeline visualizations were constructed to identify research hotspots, emerging multimodal intervention themes, and diagnostic-specific research trajectories across osteoarthritis, low back pain, chronic musculoskeletal pain, tendinopathy, and shoulder disorders. These visual knowledge maps provided an intuitive framework for examining intellectual structure, research frontiers and temporal transitions in multimodal musculoskeletal rehabilitation.

Bibliometrix (R package, version 4.3.2) was used for descriptive bibliometric analyses, thematic evolution mapping, Bradford zone analysis, and network-based data processing. Thematic evolution analysis was employed to examine the transition from unimodal rehabilitation research toward multimodal, guideline-aligned, and self-management-oriented rehabilitation approaches reflective of contemporary musculoskeletal rehabilitation practice. Altmetric data were integrated to complement traditional citation-based analyses and evaluate online research visibility and dissemination patterns.

### 2.7. Altmetric Analysis

Altmetric analysis was conducted to evaluate the digital dissemination and online attention of publications related to multimodal musculoskeletal rehabilitation. Altmetric data were retrieved for eligible publications using the Altmetric Explorer platform, which aggregates mentions from multiple online sources including social media platforms (e.g., Twitter/X; Facebook), news outlets, blogs, policy documents, and reference managers.

The Altmetric Attention Score (AAS) was used as the primary indicator of online visibility and broader societal impact. Descriptive analyses were performed to summarize the distribution of AAS values across publications and to identify highly discussed articles and thematic areas receiving public or policy attention. Altmetric indicators were examined in parallel with traditional bibliometric measures to assess the relationship between scholarly impact and digital dissemination.

Altmetric indicators, including Twitter/X mentions, Mendeley readership, and policy citations, were extracted when available to estimate digital dissemination and guideline-level attention. Policy-related mentions were of particular interest given the increasing incorporation of multimodal care pathways in musculoskeletal rehabilitation guidelines and health system initiatives. Altmetric counts were not used to compare scientific impact but rather to contextualize dissemination patterns and knowledge translation potential within multimodal rehabilitation research. Altmetric indicators were interpreted descriptively given variable metric availability and heterogeneous reporting across publication years.

### 2.8. Ethical Considerations

Ethics approval was not required as this study was based exclusively on publicly available bibliometric data.

## 3. Results

### 3.1. Annual Growth Trend of Publications

**Figure 1:**
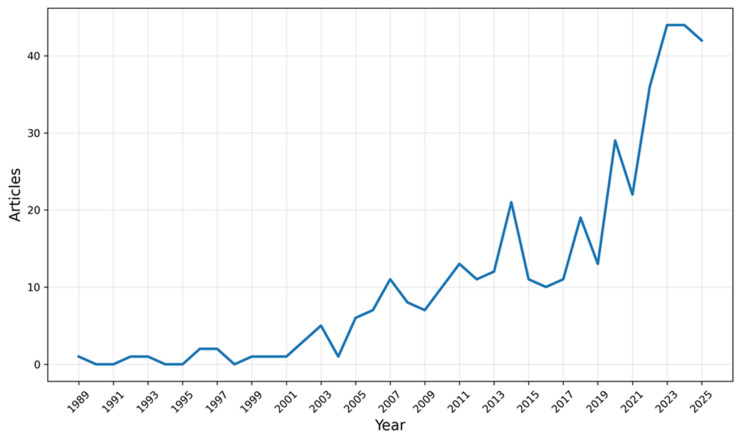
Annual publication trends in multimodal musculoskeletal rehabilitation research indexed in the Web of Science Core Collection (1989–2025). Note: Publication growth trends may partly reflect the progressive expansion of Web of Science Core Collection coverage over time.

A total of 409 publications related to multimodal musculoskeletal rehabilitation were identified in the Web of Science Core Collection. Figure 1 illustrates the annual distribution of publications across the selected timeframe. Although early records emerged in the late 1980s, scientific production remained limited throughout the 1990s. A steady increase was observed after 2000, followed by a noticeable acceleration during the past decade, reflecting growing clinical and academic interest in multimodal rehabilitation paradigms integrating exercise therapy with education, cognitive–behavioral strategies, manual therapy, or electrotherapy. This increase may partly reflect the progressive expansion and evolving indexing policies of the Web of Science Core Collection over time in addition to genuine scientific growth within multimodal rehabilitation research [33,34]. Therefore, temporal publication trends should be interpreted cautiously, particularly when comparing older and more recently indexed literature [34].

The annual growth trajectory suggests a transition from a formative exploratory phase toward a more structured and consolidating research domain. This trend parallels the increasing emphasis on guideline-aligned rehabilitation, biopsychosocial management models, and self-management frameworks in musculoskeletal disorders. The apparent decline in the most recent year is likely attributable to incomplete database indexing at the time of data extraction rather than a true decrease in scientific output.

**Figure 2:**
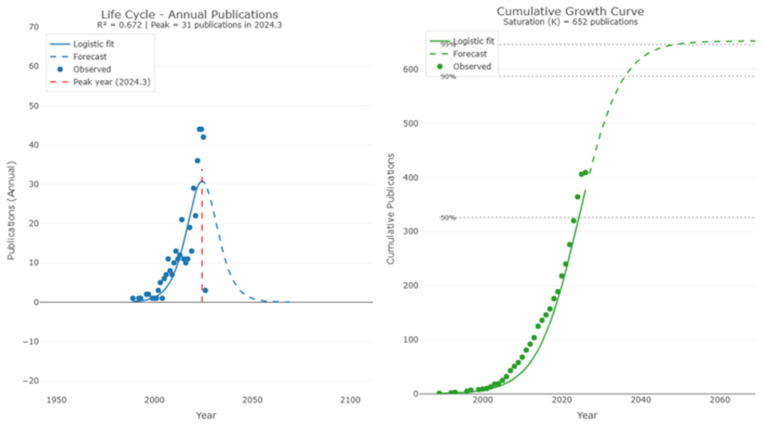
Life cycle and temporal development of scientific production in multimodal musculoskeletal rehabilitation research (1989–2026).

Beyond temporal growth, the life cycle analysis (Figure 2) demonstrated that multimodal musculoskeletal rehabilitation has progressed from an emerging research area into a developing and consolidating scientific domain characterized by increasing thematic maturity and methodological refinement.

Overall, the bibliometric dataset comprised 409 documents published across 243 scholarly sources, representing a multidisciplinary research landscape spanning rehabilitation sciences, pain medicine, musculoskeletal health, primary care, and behavioral science. The annual growth rate of 3.01% indicates a gradual and sustained expansion of the field. The mean document age of 8.35 years suggests an established research domain with a substantial historical base. The average citation rate of 48.45 citations per document further highlights meaningful scholarly impact within this research area.

Keyword analysis demonstrated high thematic variability, with 1131 Author Keywords and 1309 Keywords Plus terms, reflecting diverse multimodal intervention models and diagnostic clusters involving osteoarthritis, low back pain, chronic pain, shoulder disorders, and tendinopathies. The extensive reference pool (20,024 references) underscores the field’s scientific depth and interdisciplinary scope.

A total of 2166 authors contributed to the multimodal rehabilitation literature, with a mean of 5.91 co-authors per document, illustrating a collaborative and multidisciplinary authorship structure. International co-authorship accounted for 24.69% of publications, indicating moderate cross-country collaboration. Single-authored publications were rare (n = 27), consistent with contemporary scientific production trends in rehabilitation research.

Regarding document types, original research articles constituted the majority (n = 256), followed by review articles (n = 129), highlighting both active primary research generation and synthesis efforts in this field. Other document types (e.g., proceedings papers, editorials, and meeting abstracts) were comparatively uncommon. The main bibliometric characteristics of the dataset are summarized in Table 3.

**Figure 3:**
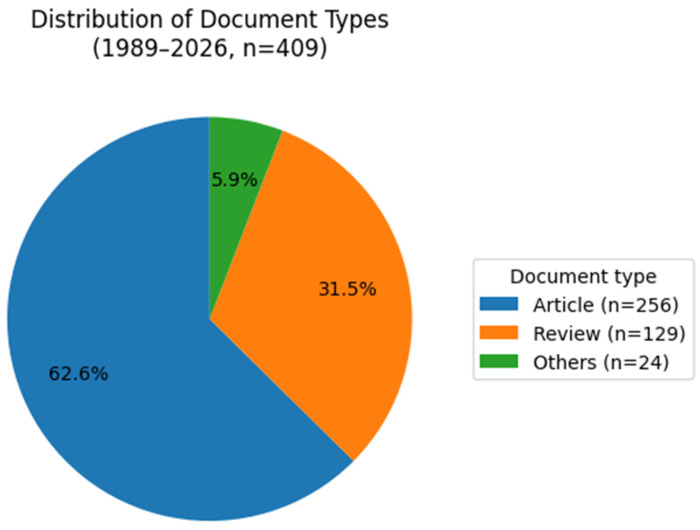
Distribution of document types in multimodal musculoskeletal rehabilitation research (1989–2026, n = 409).

Original research articles constituted the majority of publications in multimodal musculoskeletal rehabilitation (n = 256, 62.6%), followed by review articles (n = 129, 31.5%), indicating both active primary evidence generation and substantial synthesis activity in the field. Other document types (n = 24, 5.9%; including editorials, proceedings papers, early access articles, book chapters, and meeting abstracts) were comparatively uncommon (Figure 3).

### 3.2. Journals and Research Fields Analysis

**Figure 4:**
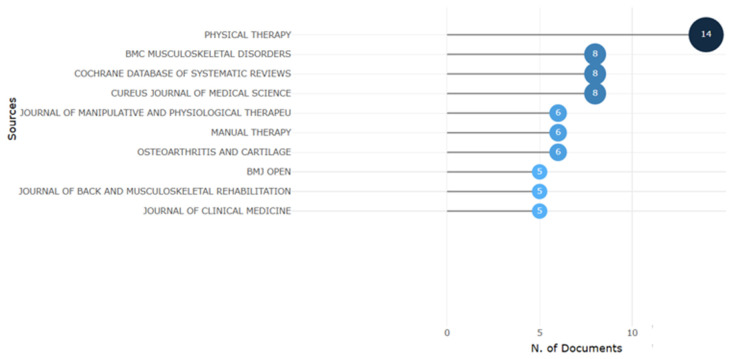
Top 10 most productive journals contributing to the multimodal musculoskeletal rehabilitation literature (1989–2026).

**Figure 5:**
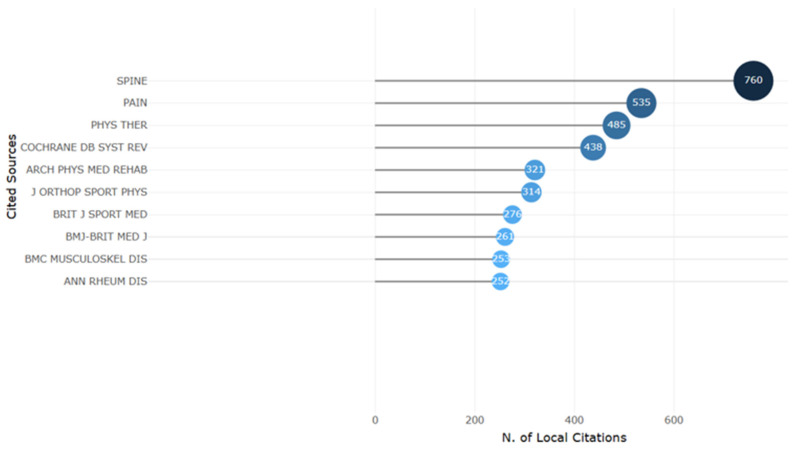
Top 10 most influential source journals identified by local citation impact in multimodal musculoskeletal rehabilitation research.

Publications related to multimodal musculoskeletal rehabilitation were distributed across a wide range of academic journals, reflecting the multidisciplinary nature of the field. In total, 243 journals contributed to the literature, spanning rehabilitation, physiotherapy, pain medicine, sports sciences, orthopedics, neurology, and general medicine. The top 10 most productive journals are presented in Figure 4 and summarized in Table 4, while Figure 5 illustrates the top 10 most influential journals based on local citation counts.

Among the most productive journals, musculoskeletal rehabilitation and physiotherapy-oriented journals such as *Journal of Back and Musculoskeletal Rehabilitation, Archives of Physical Medicine and Rehabilitation, Disability and Rehabilitation*, and *Physical Therapy & Rehabilitation Journal* ranked highly, reflecting the strong contribution of core rehabilitation outlets. Journals in sports sciences (*Journal of Sport Rehabilitation*), pain (*Pain Medicine*; *Clinical Journal of Pain*), and orthopedics (*Knee Surgery Sports Traumatology Arthroscopy*) were also represented, underscoring the cross-disciplinary relevance of multimodal rehabilitation within contemporary clinical practice.

Journal quartiles were classified according to Journal Citation Reports (JCR), where Q1 represents journals within the top 25% of their subject categories, and Q2 represents journals within the 25–50% range. Among journals with available quartile information, 168 publications (46.3%) were published in Q1 journals and 96 publications (26.4%) in Q2 journals, indicating that nearly three-quarters of the literature was concentrated in relatively high-impact and internationally visible journals.

To further evaluate journal-level influence beyond publication volume, citation-based source impact indicators including H-index, G-index, and M-index were calculated using local citation data (Table 4). Several journals demonstrated high citation impact despite moderate publication counts—particularly *Archives of Physical Medicine and Rehabilitation* and *Clinical Journal of Pain*—suggesting that methodological rigor, evidence synthesis, and guideline-relevant research output are key drivers of scholarly influence in multimodal rehabilitation rather than publication volume alone.

A considerable proportion of publications appeared in Q1–Q2 journals across Rehabilitation, Sport Sciences, Pain Medicine, and Orthopedics categories, indicating that multimodal rehabilitation research is frequently disseminated through high-impact academic outlets. Notably, several productive and influential journals operate under open access or hybrid publication models, reflecting the alignment between multimodal rehabilitation and open science dissemination, knowledge translation, and clinical implementation.

**Figure 6:**
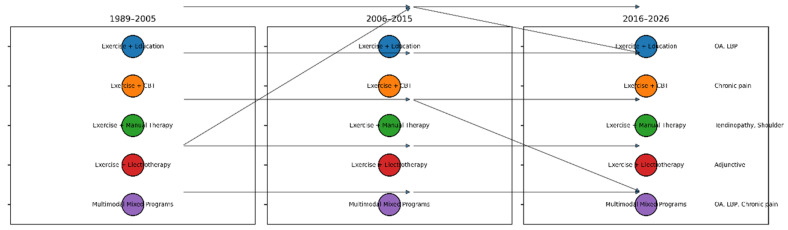
Conceptual thematic evolution of multimodal musculoskeletal rehabilitation research across three temporal phases (1989–2005, 2006–2015, and 2016–2026).

Subject category and thematic structure analyses revealed that multimodal rehabilitation spans multiple interconnected biomedical and clinical disciplines. As shown in Figure 6, core domains include rehabilitation, physiotherapy, pain medicine, sports sciences, orthopedics, neurology, and primary care. The network visualization demonstrated strong linkages among these areas, indicating high degrees of interdisciplinarity and thematic convergence. This overlap reflects the evolving biopsychosocial and guideline-based nature of multimodal rehabilitation research, which integrates exercise therapy, self-management, education, behavioral interventions, and adjunctive manual or electrotherapy-based modalities across diverse clinical and diagnostic contexts.

**Figure 7:**
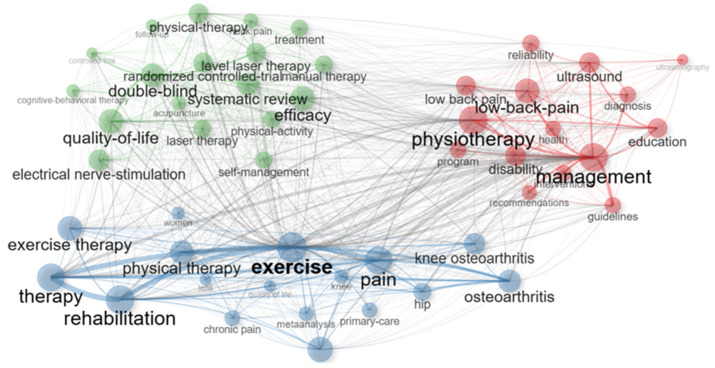
Keyword co-occurrence network and thematic clustering in multimodal musculoskeletal rehabilitation research (1989–2026) after manual normalization of synonymous keyword variants, illustrating the interdisciplinary convergence of rehabilitation, pain management, physiotherapy, and self-management-oriented care models.

Beyond journal-level patterns, cognitive field mapping identified three dominant thematic clusters (Figure 7), highlighting the multidimensional and biopsychosocial structure of multimodal rehabilitation research. These clusters encompassed (i) rehabilitation/exercise therapy, (ii) pain and musculoskeletal diagnoses, and (iii) management, guidelines, and self-management, confirming the interdisciplinary convergence of physiotherapy, rehabilitation, pain medicine, and sports sciences. This static cognitive structure aligned with the temporal evolution observed across time slices (Supplementary Figures S1–S3), demonstrating a progressive shift from unimodal biomedical approaches toward exercise-centered and guideline-aligned multimodal rehabilitation (Table 5).

To examine how these thematic structures evolved over time, the literature was divided into three temporal slices corresponding to major shifts in multimodal rehabilitation research (Figure 7 and Supplementary Figures S1–S3). Time Slice 1 (1989–2005) was characterized by unimodal biomedical interventions dominated by electrotherapy-based modalities and manual therapy. Time Slice 2 (2006–2015) represented a transitional phase marked by increasing emphasis on exercise-based rehabilitation, guideline development, and chronic musculoskeletal conditions. Time Slice 3 (2016–2026) reflected a more multimodal paradigm integrating exercise, education, and self-management strategies aligned with contemporary biopsychosocial models. These temporal patterns demonstrate a progressive shift from unimodal biomedical interventions to guideline-aligned multimodal and self-management approaches. This conceptual transition is summarized in Table 5.

Highly central nodes such as exercise therapy, education/self-management, osteoarthritis, low back pain, and cognitive behavioral therapy formed the core of the multimodal rehabilitation network, whereas tendinopathy, shoulder disorders, and electrophysical agents occupied more peripheral but clinically relevant positions. Keyword co-occurrence analysis revealed three dominant thematic clusters representing multimodal rehabilitation paradigms. A rehabilitation-centered cluster encompassed exercise therapy, education/self-management, osteoarthritis, and low back pain, aligning with guideline-based recommendations for core treatments. A second cluster comprised manual therapy and electrophysical agents associated with tendinopathy and shoulder disorders, reflecting adjunctive modalities commonly employed in sports and orthopedic rehabilitation. A third cluster emphasized cognitive behavioral therapy, chronic musculoskeletal pain, and disability measures, illustrating a biopsychosocial shift toward multimodal pain management.

Over three decades, the rehabilitation literature shifted from unimodal biomedical interventions (manual/electrophysical) to exercise-centered rehabilitation, and ultimately to guideline-aligned multimodal self-management models targeting chronic musculoskeletal pain.

**Figure 8:**
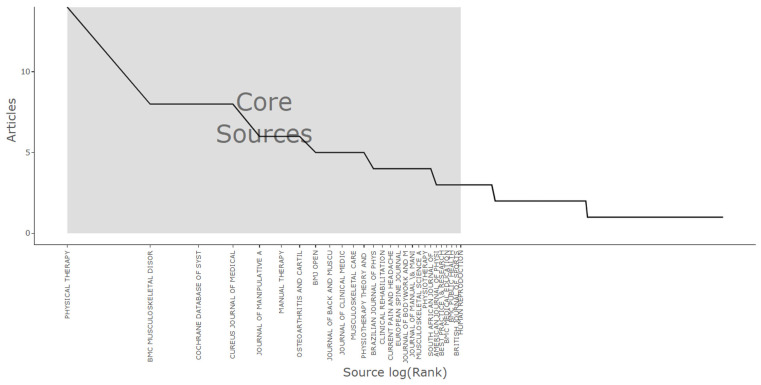
Bradford’s Law distribution of core journals in multimodal musculoskeletal rehabilitation research.

Figure 8 illustrates the distribution of publications across journals according to Bradford’s Law. A small group of core rehabilitation and pain journals accounted for a disproportionately large share of articles, forming a clearly defined Bradford core. Journals such as *Archives of Physical Medicine and Rehabilitation, Disability and Rehabilitation, Physical Therapy & Rehabilitation Journal, Pain Medicine*, and *Journal of Back and Musculoskeletal Rehabilitation* dominated the core zone, indicating their central role in disseminating multimodal rehabilitation research.

Beyond the core zone, publication output declined rapidly across a long tail of peripheral journals spanning sports sciences, orthopedics, neurology, and general medicine, reflecting the multidimensional and interdisciplinary nature of multimodal rehabilitation. This distribution aligns with the increasing emphasis on guideline-aligned, exercise-centered, and self-management-based care models in musculoskeletal rehabilitation.

The observed pattern also reflects the translational character of multimodal rehabilitation, wherein evidence synthesis and clinical practice journals jointly contribute to knowledge dissemination. Taken together, these findings confirm the applicability of Bradford’s Law to this field and identify a concentrated set of journals that serve as primary outlets for scholarly communication.

Table 6 summarizes the most productive journals publishing multimodal rehabilitation research. Rehabilitation-focused outlets (e.g., *Archives of PM&R, Disability and Rehabilitation*, and JBMR) contributed substantially, while sports medicine and pain journals (e.g., *Pain Medicine, Clinical Journal of Pain*, and KSSTA) provided complementary contributions. The presence of high-impact general clinical journals (e.g., *BMC Musculoskeletal Disorders*; *Journal of Clinical Medicine*) highlights an increasing emphasis on evidence-based rehabilitation and guideline-aligned musculoskeletal care.

Overall, the distribution of sources reflects an interdisciplinary publication footprint integrating rehabilitation, physiotherapy, pain medicine, sports sciences, and orthopedics, consistent with the biopsychosocial and multimodal orientation of contemporary musculoskeletal rehabilitation.

### 3.3. Countries/Regions and Institutions Analysis

**Figure 9:**
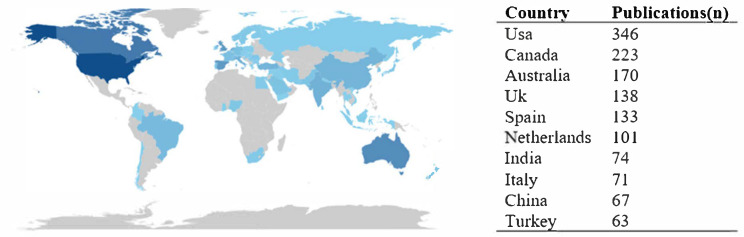
Global distribution of publications in multimodal musculoskeletal rehabilitation (1989–2026). Countries are color-coded according to publication volume, and the accompanying table lists the top ten contributing countries.

A total of 59 countries/regions contributed to the multimodal musculoskeletal rehabilitation literature, indicating a broad yet uneven global research footprint. Figure 9 illustrates the geographic distribution of publications, showing pronounced activity concentrated in North America, Western Europe, and Oceania.

The United States, Canada, Australia, and the United Kingdom formed the primary research axis, followed by Spain, the Netherlands, and Italy. These regions possess well-established rehabilitation infrastructures, high physiotherapy workforce density, and widespread implementation of guideline-driven musculoskeletal care models, which may partially explain their greater research representation within the bibliometric dataset.

Supplementary Figure S4 presents the publication output and citation impact of the top 10 most productive countries/regions. Among the leading contributors, the United States demonstrated the highest citation impact, highlighting its prominent role in scientific visibility and knowledge dissemination within multimodal musculoskeletal rehabilitation research. Other influential contributors included France and several European countries (e.g., the Netherlands and Finland), suggesting that countries with established research infrastructures may achieve substantial citation influence despite relatively lower publication volume.

The observed geographic distribution broadly parallels the global burden of musculoskeletal disorders, including osteoarthritis, low back pain, and chronic musculoskeletal pain. This pattern suggests that research activity may be partially driven by population-level rehabilitation needs, healthcare system priorities, and implementation of guideline-based rehabilitation strategies in high-burden musculoskeletal conditions.

**Figure 10:**
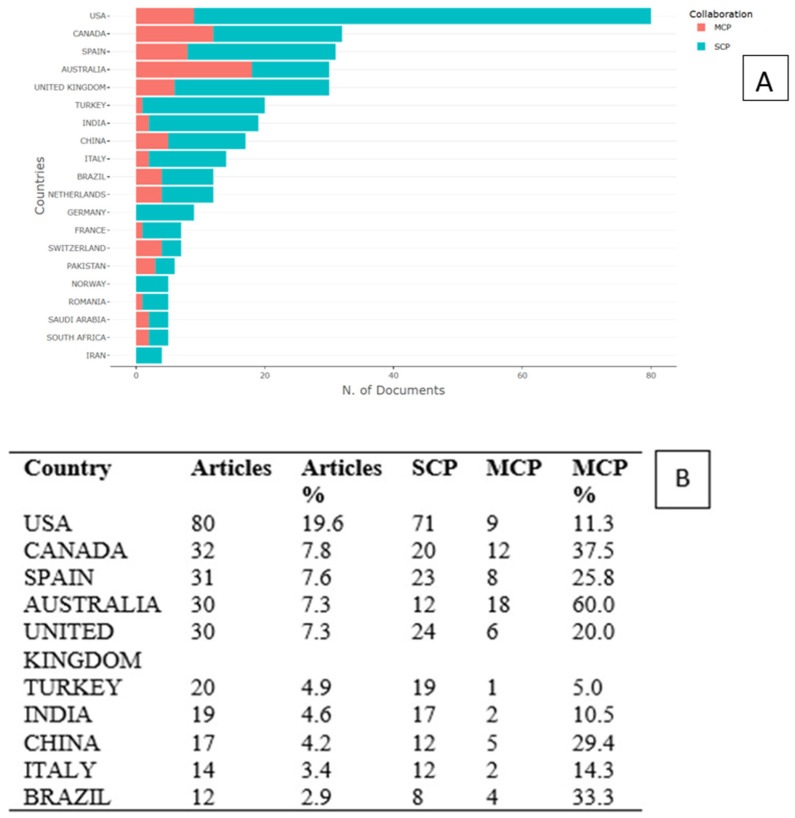
Country-level scientific production and international collaboration patterns (SCP vs. MCP) in multimodal musculoskeletal rehabilitation research. (**A**) Distribution of single-country publications (SCP) and multiple-country publications (MCP) among the most productive countries. (**B**) Tabulated summary of publication volume and SCP/MCP proportions by country, illustrating international collaboration intensity within the field.

Figure 10 displays country-level scientific production and international collaboration patterns in multimodal musculoskeletal rehabilitation research. Panel A shows the SCP–MCP distribution among the most productive countries, while Panel B summarizes corresponding proportions.

Countries such as the United States, Turkey, India, and the United Kingdom exhibited high SCP ratios, indicating predominantly domestic research networks and strong internal research infrastructures. In contrast, Australia, Canada, and Spain demonstrated markedly higher MCP ratios, reflecting stronger international collaboration and more globally integrated co-authorship networks.

The collaboration structure revealed a moderately connected global landscape, with North America, Western Europe, and Oceania forming the primary research axis. Despite increasing cross-country activity, the overall network remained partially fragmented, suggesting that international collaboration capacity and knowledge dissemination are concentrated within a limited group of high-income countries.

Panel B also highlights that MCP-oriented countries tended to achieve greater citation influence, consistent with bibliometric evidence showing that international collaboration enhances scientific visibility, research synthesis output, and guideline relevance in rehabilitation and pain medicine.

**Figure 11:**
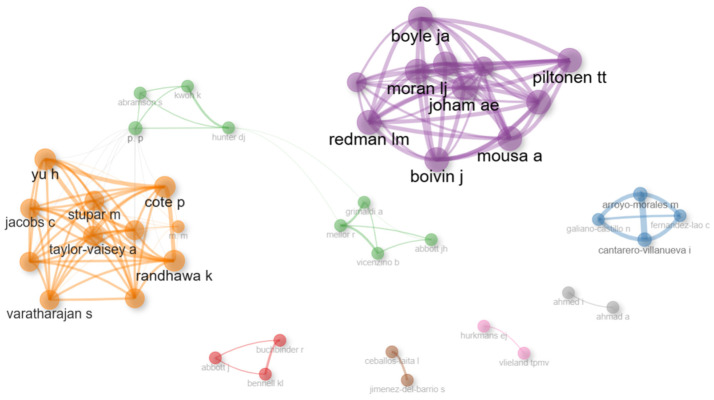
Author collaboration network in multimodal musculoskeletal rehabilitation research (1989–2026).

Figure 11 illustrates the author co-authorship network within multimodal musculoskeletal rehabilitation research. Several distinct author clusters were identified, reflecting collaborative research groups with shared thematic and methodological interests. The largest cluster was primarily associated with rehabilitation and pain-related research, characterized by strong collaboration in clinical trials and biopsychosocial rehabilitation approaches. Another prominent cluster represented physiotherapy and exercise-oriented research groups focusing on functional outcomes and physical performance measures. Smaller clusters reflected more specialized areas, including orthopedic rehabilitation and sports medicine-related research.

Although collaboration was more pronounced within individual author clusters, inter-cluster connections were also observed, indicating emerging interdisciplinary interaction across rehabilitation, physiotherapy, pain medicine, and musculoskeletal research domains. These findings suggest increasing collaborative convergence within multimodal rehabilitation research, particularly around guideline-aligned, exercise-centered, and self-management-oriented approaches. This pattern is consistent with the broader thematic evolution identified in the field, in which biopsychosocial and multidisciplinary rehabilitation models have progressively replaced unimodal biomedical approaches.

**Figure 12:**
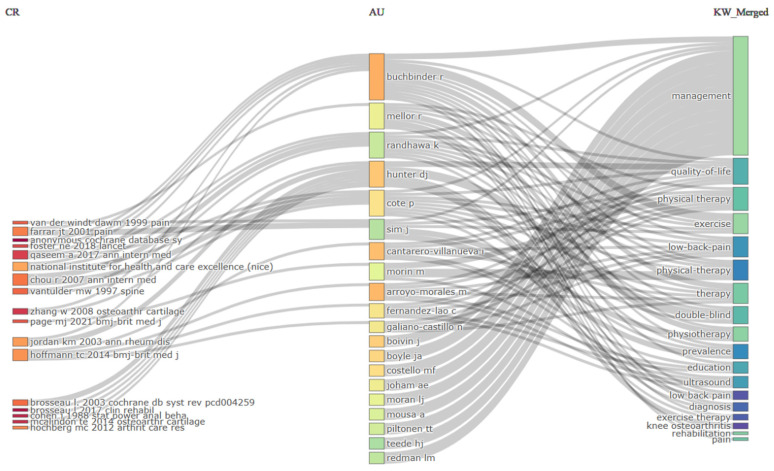
Three-field plot linking influential cited references (CR), leading authors (AU), and author keywords (KW) in multimodal musculoskeletal rehabilitation research (1989–2026).

Figure 12 presents a three-field plot linking influential cited references, leading authors, and merged author keywords in multimodal musculoskeletal rehabilitation research. The left field highlights a concentrated set of foundational evidence sources, dominated by systematic reviews, clinical guidelines, and landmark trials, indicating a strong evidence-synthesis and guideline-oriented foundation for the field.

The central field identifies a core group of prolific authors who act as key intellectual connectors across thematic areas, contributing to multiple musculoskeletal conditions and rehabilitation approaches rather than a single research niche. This structure reflects a diversified authorship pattern consistent with multimodal, guideline-informed rehabilitation research.

The right field reveals that clinical and rehabilitation-oriented keywords form the dominant thematic space, encompassing exercise therapy, physiotherapy, education/self-management, functional disability, and pain-related outcomes. Condition-specific terms such as osteoarthritis and low back pain further indicate that multimodal rehabilitation research is anchored in chronic musculoskeletal disorders with high disease burden and guideline relevance.

Together, the three-field plot demonstrates a coherent cognitive structure in which foundational evidence sources inform a relatively stable group of leading authors, who, in turn, drive clinically relevant research themes aligned with biopsychosocial and self-management-oriented models of musculoskeletal rehabilitation. This cognitive structure aligns with the observed thematic evolution from unimodal biomedical models toward guideline-aligned multimodal self-management approaches.

### 3.4. Analysis of Authors and Co-Cited Authors

**Figure 13:**
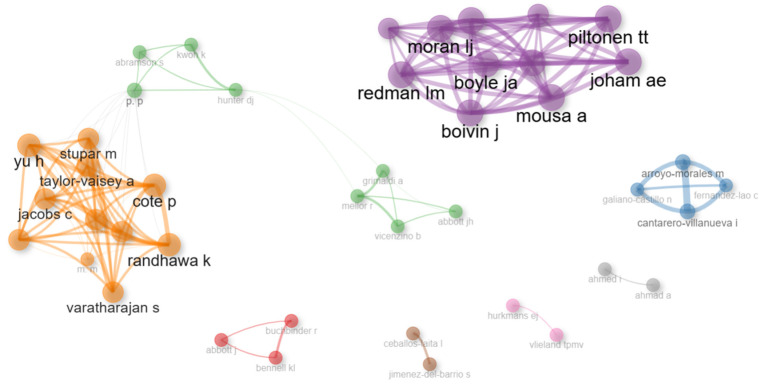
Co-authorship network of authors in multimodal musculoskeletal rehabilitation research (1989–2026).

**Figure 14:**
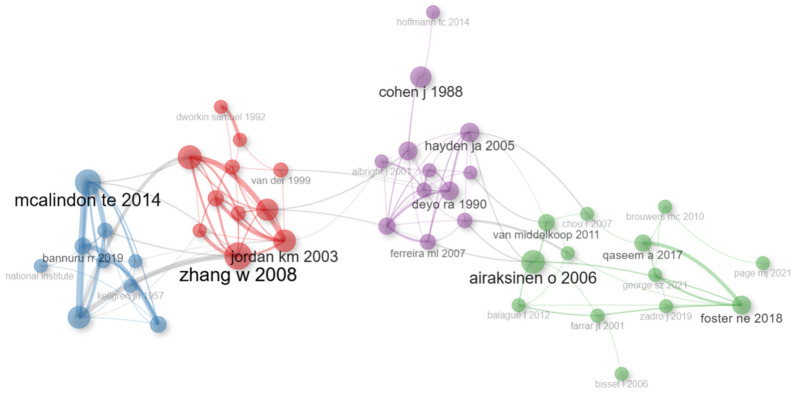
Author co-citation network in multimodal musculoskeletal rehabilitation research (1989–2026).

A total of 2166 authors contributed to multimodal musculoskeletal rehabilitation research during the study period (1989–2026). Author productivity and scholarly influence were examined using publication-based metrics, local citation performance, and network-based indicators. To characterize the intellectual and collaborative structure of the field, author-level co-authorship and co-citation networks were visualized (Figure 13 and Figure 14). In addition, the top 10 most productive authors and the top 10 most highly co-cited authors are summarized in Table 7 and Table 8.

Author productivity and scholarly influence exhibited a partially decoupled pattern. Table 7 shows that Buchbinder R., Arroyo-Morales M., Cantarero-Villanueva I., and Hunter D.J. ranked among the most productive authors based on publication counts and fractionalized authorship, indicating sustained output within rehabilitation and chronic musculoskeletal pain research domains.

In contrast, citation-based influence was concentrated around a different set of contributors. As shown in Table 8, Cedraschi C., Airaksinen O., Brox J.I., Klaber-Moffett J., Kovacs F., and related guideline-oriented contributors exhibited the highest local citation counts, reflecting the enduring intellectual impact of evidence synthesis, guideline development, and chronic pain-focused research.

Co-authorship analysis revealed a moderately collaborative landscape, with an average of 5.91 co-authors per document and 24.69% international collaboration, indicating that research is primarily conducted through multi-authored teams with partial engagement in cross-country networks. These values are consistent with other rehabilitation-oriented domains, indicating moderate collaboration density and partial internationalization. A small proportion of single-authored documents (n = 27) suggests that multimodal rehabilitation research is not predominantly driven by individual scholarship but rather by collaborative clinical and methodological groups.

Co-citation network analysis demonstrated strong intellectual interconnections among highly cited authors, suggesting a shared evidence base and frequent cross-referencing across guideline-aligned and biopsychosocial rehabilitation themes. Together, these findings indicate a collaborative yet partially segmented authorship structure in which key contributors anchor thematic subdomains aligned with biopsychosocial, guideline-informed, and self-management-oriented models of multimodal rehabilitation.

### 3.5. Analysis of Keywords

**Figure 15:**
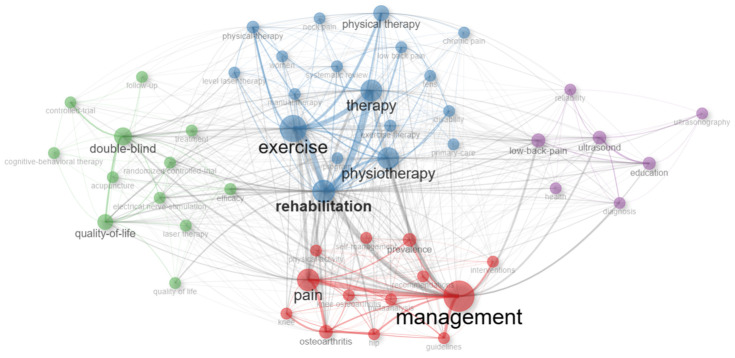
Keyword co-occurrence network based on keyword frequency in multimodal musculoskeletal rehabilitation research (1989–2026).

Figure 15 displays the keyword co-occurrence network of multimodal musculoskeletal rehabilitation research. High-frequency terms included rehabilitation, exercise, physiotherapy, therapy, pain, management, osteoarthritis, low back pain, and self-management, indicating that research activity converges on guideline-aligned multimodal rehabilitation for chronic musculoskeletal disorders.

Three dominant thematic clusters emerged:

(1) A rehabilitation/exercise/physiotherapy cluster, reflecting core multimodal treatment modalities;
(2) A pain/management/osteoarthritis/low back pain cluster, representing high-burden musculoskeletal conditions;
(3) A clinical evidence cluster centered on randomized controlled trials, quality-of-life outcomes, and self-management.

Betweenness centrality highlighted rehabilitation, exercise therapy, pain, and management as influential bridging keywords linking treatment modalities with chronic musculoskeletal conditions, suggesting a coherent, biopsychosocial, and self-management-oriented thematic structure consistent with contemporary rehabilitation guidelines.

**Figure 16:**
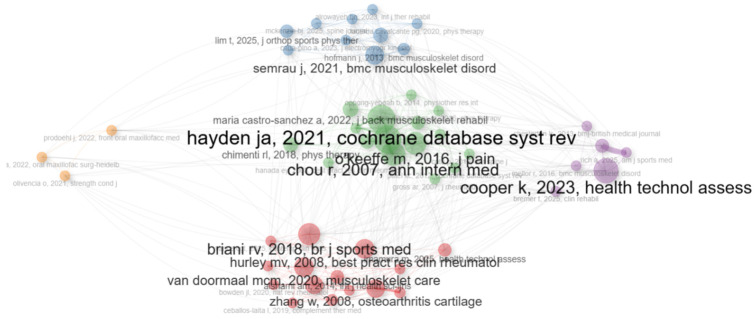
Thematic clustering of highly cited references (LLR) revealing major evidence domains in multimodal musculoskeletal rehabilitation research (1989–2026).

Figure 16 illustrates the thematic clustering of highly cited references using the log-likelihood ratio (LLR) algorithm, identifying major evidence domains in multimodal musculoskeletal rehabilitation research. Major clusters reflected evidence synthesis and guideline-oriented outputs (e.g., Cochrane reviews, health technology assessments, and systematic reviews), chronic musculoskeletal pain and osteoarthritis, physiotherapy and exercise-based rehabilitation, and sports medicine and functional outcomes. Together, these clusters indicate that the intellectual foundation of multimodal rehabilitation research is anchored in high-burden chronic musculoskeletal conditions and supported by rigorous evidence frameworks.

A prominent cluster consisted of guideline- and evidence-synthesis-oriented publications focusing on osteoarthritis and chronic musculoskeletal pain, underscoring the role of evidence-based evaluation in shaping rehabilitation recommendations and clinical practice. Another major cluster was centered on rehabilitation and physiotherapy trials addressing exercise therapy, functional recovery, disability, and quality-of-life outcomes, representing the applied clinical dimension of multimodal rehabilitation research.

More recent clusters emphasized biopsychosocial and self-management-aligned rehabilitation models incorporating education, cognitive–behavioral components, and guideline-informed intervention programs. Their temporal recency suggests ongoing thematic evolution toward holistic and patient-centered rehabilitation approaches.

Collectively, the keyword and reference clustering analyses demonstrate a progressive transition from early exploratory research toward outcome-oriented, evidence-based, and guideline-aligned models of multimodal musculoskeletal rehabilitation. This maturation pattern is consistent with the broader thematic evolution observed in Figure 6 and Figure 7, Supplementary Figures S1–S3, and the ongoing shift toward biopsychosocial and guideline-informed rehabilitation approaches.

**Figure 17:**
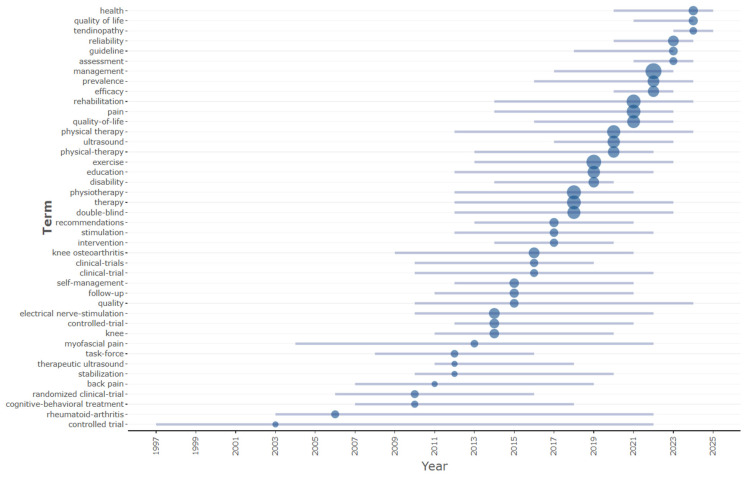
Temporal evolution of author keywords in multimodal musculoskeletal rehabilitation research (1989–2026).

Temporal keyword analysis revealed a progressive shift in research focus across the study period (Figure 17). Early publications (1990s–early 2000s) predominantly emphasized methodological and trial-related terms (e.g., randomized clinical trial, controlled trial, task-force, and placebo), reflecting a biomedical and efficacy-oriented phase of multimodal rehabilitation research.

From the mid-2000s to late 2010s, the thematic emphasis transitioned toward core rehabilitation constructs (exercise, physiotherapy, rehabilitation, disability, and quality of life) alongside condition-specific terms (low back pain; osteoarthritis). This period corresponds to the integration of exercise-centered and biopsychosocial rehabilitation models within musculoskeletal care.

In the most recent decade (2018–2026), emergent terms such as self-management, guideline, management, prevalence, education, ultrasound, and reliability became increasingly prominent, indicating a consolidation toward guideline-aligned, outcome-focused, and self-management-oriented rehabilitation paradigms. The late emergence of task-specific diagnostic and assessment terms reflects enhanced methodological refinement and clinical implementation.

Overall, the temporal trajectory highlights a structured evolution from unimodal, trial-based biomedical interventions toward multimodal, biopsychosocial, and guideline-informed rehabilitation approaches with emphasis on functional outcomes and self-management in chronic musculoskeletal disorders. This temporal pattern parallels the conceptual thematic evolution described in Figure 6, showing a shift from unimodal biomedical treatments toward multimodal, guideline-aligned, and self-management-oriented rehabilitation models.

### 3.6. Co-Cited References and Highly Cited Papers

**Figure 18:**
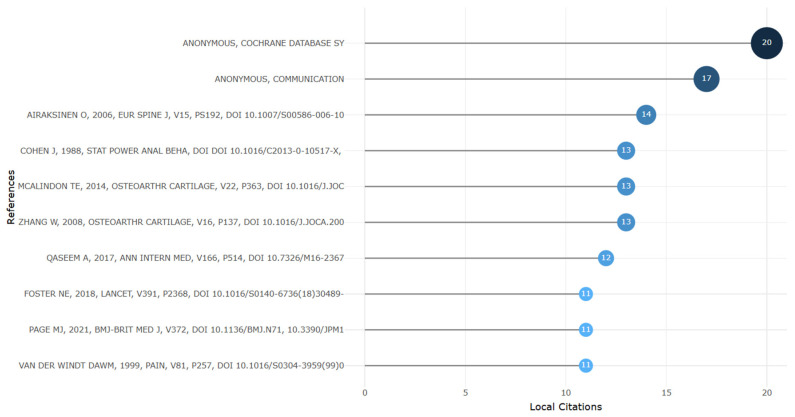
Top 10 most locally cited documents in multimodal musculoskeletal rehabilitation research (1989–2026).

Citation analyses were conducted to identify the most influential publications shaping the intellectual foundation of multimodal musculoskeletal rehabilitation. Figure 18 presents the top 10 locally cited documents, reflecting publications that exert strong influence within the analyzed corpus. These studies predominantly consist of exercise- and physiotherapy-based clinical trials, systematic reviews, and guideline documents addressing chronic musculoskeletal pain conditions such as low back pain and osteoarthritis—conditions that constitute the primary targets of multimodal rehabilitation.

**Figure 19:**
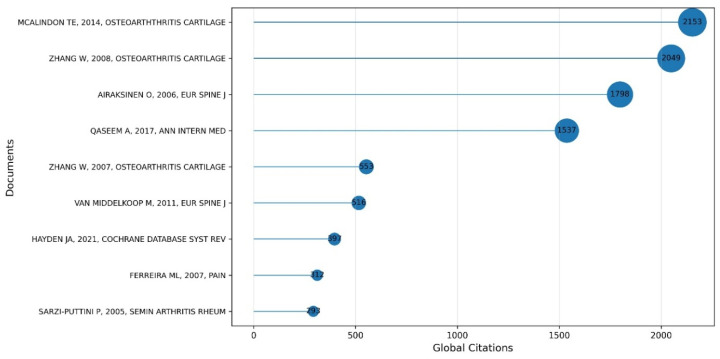
Top nine most globally cited documents in multimodal musculoskeletal rehabilitation research (1989–2026).

Figure 19 displays the top nine globally cited documents, demonstrating broader scholarly influence beyond the dataset itself. These highly cited works are largely evidence-synthesis outputs (e.g., OARSI recommendations, European guidelines, and ACP guidelines) and high-impact clinical trials informing chronic pain management and exercise-based rehabilitation. The overlap between locally and globally highly cited publications confirms that multimodal rehabilitation research is methodologically anchored in guideline-aligned, evidence-based frameworks.

Table 9 summarizes the top globally cited clinical evidence and guideline papers, illustrating that the intellectual structure of multimodal rehabilitation is grounded in authoritative guideline documents, systematic reviews, and large-scale clinical trials. These works emphasize exercise therapy, nonsurgical management, functional recovery, and chronic musculoskeletal pain—core pillars of multimodal rehabilitation.

**Figure 20:**
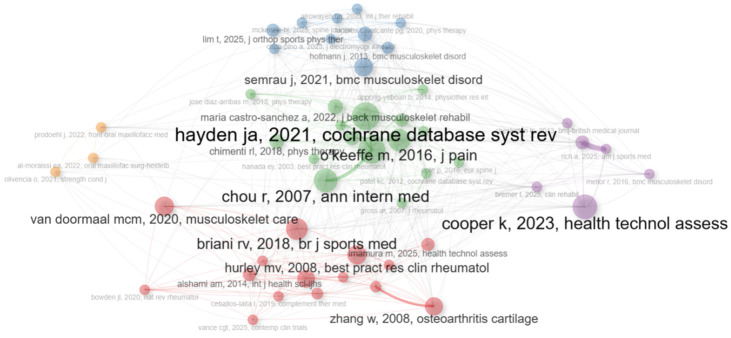
Bibliographic coupling network based on shared reference patterns (1989–2026), revealing major thematic clusters in multimodal musculoskeletal rehabilitation research. Clusters reflect evidence synthesis and guidelines, physiotherapy and exercise-based rehabilitation, sports medicine and osteoarthritis, and emerging self-management–oriented approaches.

Figure 20 presents the bibliographic coupling network based on shared reference patterns, identifying coherent thematic clusters. Bibliographic coupling revealed that multimodal rehabilitation research is structured around several dominant research streams:

- Evidence-synthesis and guideline cluster, anchored by OARSI, ACP, and European recommendations, systematic reviews, and HTA outputs providing the methodological and epistemic foundation for multimodal care;
- Exercise- and physiotherapy-centered clinical trial cluster, emphasizing rehabilitation outcomes, disability reduction, quality of life, and functional recovery.
- Condition-specific cluster, focused on high-burden chronic musculoskeletal conditions such as osteoarthritis, chronic low back pain, and tendinopathies;
- Emerging biopsychosocial/self-management cluster, characterized by education, behavioral components, and guideline-aligned self-management interventions.

The network demonstrates dense intra-cluster connectivity and weaker inter-cluster links, suggesting that multimodal rehabilitation research is organized around well-defined thematic streams rather than fragmented outputs—consistent with the conceptual maturation of the field.

Table 10 lists the top locally highly co-cited references, representing the intellectual core of multimodal musculoskeletal rehabilitation. These foundational papers—mostly guidelines, systematic reviews, and physiotherapy-based rehabilitation trials—have served as key knowledge inputs shaping chronic musculoskeletal rehabilitation practice.

Collectively, the citation, co-citation, and bibliographic coupling analyses suggest a progressive thematic transition from unimodal biomedical approaches toward more integrated and evidence-informed multimodal rehabilitation frameworks.

Overall, the citation-based intellectual structure indicates that multimodal musculoskeletal rehabilitation has transitioned from unimodal biomedical interventions toward guideline-aligned, biopsychosocial, and self-management-oriented models supported by authoritative evidence syntheses and condition-specific clinical trials.

## 4. Discussion

### 4.1. General Information

The growing global burden of musculoskeletal pain and functional disability has intensified the need for rehabilitation approaches that extend beyond unimodal biomedical interventions toward multimodal, biopsychosocial, and self-management-oriented care models. The citation- and co-citation-based intellectual structure identified in this study confirms that multimodal musculoskeletal rehabilitation has progressively transitioned from unimodal biomedical interventions toward guideline-aligned, biopsychosocial, and self-management-oriented models. This pattern is reflected in the prominence of authoritative guideline documents (e.g., OARSI, ACP, and European low back pain guidelines) and physiotherapy-based clinical trials addressing exercise therapy, disability, functional recovery, and pain outcomes.

The alignment between the bibliometric evidence structure and contemporary clinical guidelines supports the view that multimodal rehabilitation has increasingly emerged as a major framework within contemporary musculoskeletal care. This coherence reinforces the clinical applicability and implementation relevance of multimodal rehabilitation models and is consistent with recommendations prioritizing exercise therapy, education/self-management, cognitive–behavioral components, and adjunctive physiotherapy interventions as first-line treatment strategies for chronic low back pain and osteoarthritis.

Publication output in multimodal musculoskeletal rehabilitation has increased steadily over the past three decades, particularly after 2000, paralleling the rising global burden of chronic musculoskeletal disorders and associated disability [1,2,3,4]. To our knowledge, this study represents the first comprehensive bibliometric analysis mapping the intellectual structure, research hotspots, and thematic evolution of multimodal rehabilitation, integrating exercise therapy, physiotherapy, self-management, and guideline-aligned care models [5,6,8,9,13,16,17].

This trend is consistent with contemporary global health frameworks recognizing musculoskeletal disorders as a major cause of disability and a priority area for health system development [1,2,3,4]. The observed thematic shift toward biopsychosocial, guideline-aligned, exercise-centered, and self-management-oriented care models reflects strong alignment with evidence-based clinical recommendations for chronic musculoskeletal pain and osteoarthritis [9,13,14,16,17,21,24,25,36,37,38,39,40,41,42,43].

Research in this field has been disseminated across a broad range of rehabilitation, musculoskeletal, and pain medicine journals, underscoring its multidisciplinary character. High-income countries with established physiotherapy infrastructures and guideline-driven MSK care models—such as the United States, Canada, Australia, the United Kingdom, and the Netherlands—emerged as major contributors, reflecting the close relationship between research capacity, clinical service delivery models, and guideline implementation [5,8,40,42,43].

At the authorship level, productivity and citation influence appeared partially decoupled; prolific contributors were primarily associated with clinical rehabilitation research, whereas the most highly co-cited authors aligned with guideline and evidence-synthesis outputs [9,13,14,21,36,41]. This dual structure indicates that multimodal rehabilitation is driven by both clinical trials addressing functional outcomes and authoritative evidence frameworks informing practice recommendations.

Overall, the bibliometric findings demonstrate a conceptual and thematic evolution from unimodal biomedical interventions toward multimodal, guideline-aligned, biopsychosocial, and self-management-oriented rehabilitation paradigms [5,6,7,18,19,20,24,25,36,37,41,42,43], consistent with modern rehabilitation science and chronic pain management frameworks.

Taken together, these findings contextualize the scientific maturation of multimodal musculoskeletal rehabilitation and provide the framework for interpreting the emerging thematic patterns described in the subsequent section.

### 4.2. Global Trends and Research Hotspots

This bibliometric analysis mapped nearly four decades of multimodal musculoskeletal rehabilitation research and demonstrated a clear thematic evolution from unimodal biomedical interventions toward multimodal, guideline-aligned, biopsychosocial, and self-management-oriented care models. This pattern mirrors contemporary rehabilitation frameworks recognizing chronic musculoskeletal pain as a complex condition requiring multidimensional management strategies [18,19,20,36,37].

Across time, exercise consistently emerged as the foundational rehabilitation modality, forming the core around which adjunctive physiotherapy interventions, manual therapy techniques, and behavioral/self-management strategies were organized. This aligns with guideline recommendations for osteoarthritis and chronic low back pain, in which exercise and education constitute core interventions [9,13,14,16,17].

A key trend was the progressive shift away from passive electrophysical agents and manual therapy monotherapy toward exercise-centered multimodal programs incorporating education, functional training, and cognitive–behavioral components. This transition is consistent with international guideline statements emphasizing non-pharmacological, exercise-based, and behavioral interventions as first-line treatments for chronic musculoskeletal pain and disability [9,13,14,15,16,17,21,24,25,36,37].

Thematic clustering further revealed the coexistence of two dominant knowledge streams: (i) evidence-synthesis and guideline frameworks and (ii) rehabilitation and physiotherapy clinical trials. Together, these streams reflect the dual structure of multimodal rehabilitation as both an evidence-driven and clinically operationalized paradigm. The dense intra-cluster connectivity observed within these streams suggests methodological maturity and conceptual coherence, while weaker inter-cluster connections highlight opportunities for greater integration across exercise, behavioral and physiotherapy-based approaches [21,24,25,36,37,41,42,43].

From a research standpoint, the predominance of exercise as a core treatment modality underscores its foundational role within musculoskeletal rehabilitation [21,22,23] while also raising unresolved questions regarding optimal adjunctive combinations, sequencing, dosing, and patient stratification. From a clinical and implementation perspective, the observed thematic evolution supports condition-specific multimodal rehabilitation pathways that emphasize functional recovery, patient activation, and self-management—principles central to modern chronic pain and osteoarthritis management [9,13,14,15,16,17,18,19,20,36,37].

Finally, these findings highlight emerging research priorities for multimodal rehabilitation, including pragmatic trial designs, implementation science frameworks, digital and tele-rehabilitation models, and the incorporation of real-world evidence to support guideline adoption and care delivery within health systems [38,39,40,41,42,43]. Such directions are consistent with ongoing calls to advance patient-centered chronic pain rehabilitation and bridge the translational gap between research evidence and clinical practice [18,19,20,36,37,40,41,42].

### 4.3. Clinical Implications

The thematic and diagnostic patterns observed in this analysis reflect contemporary rehabilitation practice, in which exercise, education, and behavioral strategies constitute core components for chronic musculoskeletal disorders. For conditions such as osteoarthritis, chronic low back pain, and chronic pain, multimodal rehabilitation aligns with guideline-recommended first-line interventions emphasizing self-management, functional recovery, and patient activation rather than passive modalities. In tendinopathy and shoulder disorders, multimodal programs incorporating exercise and manual therapy correspond with impairment-focused rehabilitation models used in clinical settings. The predominance of multimodal care suggests that rehabilitation providers are increasingly integrating biopsychosocial frameworks into everyday practice while maintaining condition-specific treatment pathways. These findings support the relevance of multimodal rehabilitation for routine clinical care, health services planning, and training of rehabilitation professionals.

### 4.4. Advantages and Limitations

Strengths

- This is the first bibliometric analysis to characterize multimodal rehabilitation, addressing a gap within the literature that has focused on isolated modalities or single diagnoses;
- The study integrates bibliometric, thematic, diagnostic, and altmetric dimensions, offering a multidomain perspective on rehabilitation science;
- The findings contextualize multimodal research within contemporary guidelines, biopsychosocial models, and practice pathways, enhancing clinical relevance;
- Diagnostic subgrouping provides insight into condition-specific rehabilitation strategies, bridging research and clinical practice.

Limitations

- Bibliometric analyses are dependent on database indexing and may underrepresent non-indexed or grey literature;
- The Web of Science Core Collection was selected because of its methodological consistency, structured citation indexing, and compatibility with bibliometric mapping tools. However, other databases such as Scopus and PubMed may provide broader journal coverage and yield additional records;
- The search strategy prioritized highly specific multimodal rehabilitation combinations and therefore may have underrepresented certain multidisciplinary and integrative rehabilitation approaches, including yoga-based rehabilitation models and other mind–body rehabilitation frameworks. Emerging evidence suggests that such interventions may also function as clinically relevant multimodal rehabilitation frameworks by integrating exercise, breathing techniques, behavioral modification, body awareness, and patient-centered self-management strategies [36,37,38];
- Previous bibliometric studies have highlighted potential limitations of the Web of Science Core Collection related to metadata inconsistencies, citation indexing variability, database-dependent coverage bias, and historical coverage expansion. Because the coverage and indexing policies of the Web of Science Core Collection have evolved over time, historical bibliometric trends should be interpreted with consideration of potential database-dependent variations in retrospective coverage and indexing practices. These factors may influence long-term bibliometric trends, citation-based analyses, and thematic mapping results, particularly for older literature from the 1980s and early 1990s [34,36];
- In addition, the progressive expansion and evolving indexing policies of the Web of Science Core Collection over time may have influenced the observed publication growth trends and the apparent decline in the most recent years because of incomplete indexing at the time of data extraction. Therefore, temporal increases in publication output may partly reflect database expansion in addition to genuine scientific growth within the field;
- Altmetric data were available for only a subset of studies and may not fully reflect research translation or clinical uptake;
- The analysis focused on musculoskeletal conditions and may not generalize to neurological or cardiorespiratory rehabilitation domains;
- Clinical outcomes, treatment efficacy, and comparative effectiveness were beyond the scope of this bibliometric design and warrant future empirical investigation.

### 4.5. Future Research Directions

This bibliometric and altmetric analysis indicates that multimodal, guideline-informed, and biopsychosocial rehabilitation models now represent the dominant paradigm in musculoskeletal care. Future research should further clarify how multimodal interventions can be optimized, sequenced, and tailored across diagnostic subgroups such as osteoarthritis, low back pain, and chronic musculoskeletal pain, where exercise, education, and behavioral strategies are considered core components [9,13,14,15,16,17,18,19,20,36,37].

Pragmatic multimodal trial designs and real-world evidence will be essential to reflect actual rehabilitation delivery and patient trajectories in routine practice [41,42,43]. Implementation science frameworks may accelerate the translation of guideline-based rehabilitation into health systems, particularly for self-management and behavioral care pathways that require sustained patient engagement [18,19,20,36,37]. Emerging digital and tele-rehabilitation models also warrant further investigation given their potential to enhance accessibility, scalability, and continuity of care for chronic pain and osteoarthritis populations [38,39,40]. Integration of outcome measurement, patient stratification, and reimbursement structures may further support the clinical adoption and sustainability of multimodal rehabilitation within musculoskeletal health systems [40,42,43]. Incorporation into pre-licensure training and continuing professional development may additionally strengthen workforce capacity and implementation fidelity. Future bibliometric studies may also benefit from incorporating broader multidisciplinary and mind–body rehabilitation approaches, including yoga-based and integrative rehabilitation models, to better reflect the diversity of contemporary multimodal musculoskeletal rehabilitation practice. Recent studies investigating yoga-based rehabilitation interventions for musculoskeletal disorders further support the growing role of integrative and mind–body approaches within contemporary multimodal rehabilitation paradigms [36,37,38].

From an implementation perspective, multimodal rehabilitation aligns with current delivery models in musculoskeletal care, which emphasize self-management support, functional recovery, and chronic care pathways. Embedding multimodal principles into rehabilitation training and continuing professional development may enhance workforce readiness and implementation fidelity. Such alignment between research, training, and health systems also supports the scalability and sustainability of multimodal rehabilitation in routine clinical practice.

## 5. Conclusions

This study provides a comprehensive bibliometric and altmetric overview of multimodal musculoskeletal rehabilitation research. Our findings demonstrate that multimodal care—anchored in exercise and supported by manual therapy, electrotherapy, education, and cognitive–behavioral components— has increasingly emerged as a major scientific and clinical framework within contemporary musculoskeletal rehabilitation. Thematic evolution and diagnostic subgroup analyses revealed substantial alignment between research activity and guideline recommendations for osteoarthritis, low back pain, and chronic pain, underscoring the integration of biopsychosocial and self-management models within contemporary rehabilitation practice [9,13,14,15,16,17,18,19,20,36,37].

From a policy and implementation perspective, multimodal rehabilitation has relevance for health system planning, chronic pain policy, and musculoskeletal care reform, particularly given the rising global burden of disability associated with musculoskeletal disorders [1,2,3,4]. Future rehabilitation frameworks may benefit from pragmatic multimodal trials, real-world evidence, and digital rehabilitation innovations to enhance adoption, equity, and sustainability across diverse care settings [38,39,40,41,42,43]. Collectively, these findings support ongoing efforts to embed guideline-informed and self-management-oriented rehabilitation pathways within musculoskeletal health systems.

## Figures and Tables

**Table 1:** Search strategy and results.

Study	Data Source	Retrieval Strategy	Publications
#1	Web of Science Core Collection–Science Citation Index Expanded (SCI-EXPANDED; coverage: 1900–present) and Social Sciences Citation Index (SSCI; coverage: 1956–present)	Topic = (“exercise” OR “exercise therapy” OR “therapeutic exercise” OR physiotherapy OR “physical therapy”) AND (“manual therapy” OR “mobilization” OR “mobilisation” OR “manipulation” OR “spinal manipulation”) AND (“electrotherapy” OR “TENS” OR “ultrasound” OR “laser” OR “shockwave” OR “ESWT”) AND (“education” OR “self-management” OR “self management” OR “cognitive behavioral” OR “CBT”)	409
		Document types: Article OR Review Article	
		Language: English	
		Timespan: Database inception–10 January 2026	

**Table 2:** Main multimodal rehabilitation intervention domains.

Intervention Modality	Description	Representative Diagnostic Indications
Exercise + Education	Exercise therapy combined with patient education and self-management strategies	Osteoarthritis; Low back pain
Exercise + Cognitive–Behavioral Therapy	Exercise therapy integrated with behavioral or cognitive–functional interventions targeting pain, fear, avoidance, or coping	Chronic musculoskeletal pain
Exercise + Manual Therapy	Exercise therapy combined with hands-on techniques such as mobilization or manipulation	Tendinopathy; Shoulder disorders
Exercise + Electrotherapy	Exercise therapy integrated with electrophysical agents (e.g., TENS, ultrasound, ESWT, and laser)	Tendinopathy; Shoulder disorders (adjunctive)
Multimodal Mixed Programs	Rehabilitation programs combining exercise with two or more adjunct modalities (manual, electrotherapy, education, and behavioral)	Chronic pain; Osteoarthritis; Low back pain

Abbreviations: TENS: Transcutaneous Electrical Nerve Stimulation; ESWT: Extracorporeal Shockwave Therapy.

**Table 3:** Main bibliometric characteristics of the dataset.

**Main Information About Data**	
Timespan	1989:2026
Sources (Journals, Books, etc.)	243
Documents	409
Annual Growth Rate %	3.01
Document Average Age	8.35
Average citations per doc	48.45
References	20,024
**Document Contents**	
Keywords Plus (ID)	1309
Author’s Keywords (DE)	1131
**Authors**	
Authors	2166
Authors of single-authored docs	25
**Authors Collaboration**	
Single-authored docs	27
Co-Authors per Doc	5.91
International co-authorships %	24.69
**Document Types**	
Article	256
Article; book chapter	1
Article; early access	6
Article; proceedings paper	5
Editorial material	5
Meeting abstract	1
Proceedings paper	6
Review	129

**Table 4:** Local citation impact of source journals in multimodal musculoskeletal rehabilitation research (1989–2026).

Source	H_Index	G_Index	M_Index	TC	NP	PY_Start
*Physical Therapy*	11	14	0.324	998	14	1993
*Cochrane Database of Systematic Reviews*	8	8	0.421	1011	8	2008
*BMC Musculoskeletal Disorders*	7	8	0.389	165	8	2009
*Journal of Manipulative and Physiological Therapeutics*	6	6	0.250	125	6	2003
*Manual Therapy*	6	6	0.300	364	6	2007
*Osteoarthritis and Cartilage*	6	6	0.300	4875	6	2007
*Brazilian Journal of Physical Therapy*	4	4	0.211	30	4	2008
*Cureus Journal of Medical Science*	4	7	0.800	55	8	2022
*European Spine Journal*	4	4	0.190	2610	4	2006
*Journal of Clinical Medicine*	4	5	0.800	59	5	2022

Abbreviations: H-Index, Hirsch index; G-Index, Egghe index; M-Index, m quotient (H-Index normalized by publication period); TC, total citations; NP, number of publications; PY_Start, publication year start.

**Table 5:** Shift from unimodal interventions to multimodal self-management paradigms.

Phase	Dominant Mode	Domain
Time Slice 1	Unimodal	Electro + Manual + OA
Time Slice 2	Transitional	Exercise + Rehab + Guidelines
Time Slice 3	Multimodal	Self-Management + Exercise + Rehab

Abbreviations: OA, osteoarthritis; Rehab, rehabilitation.

**Table 6:** Most productive journals in multimodal musculoskeletal rehabilitation (1989–2026).

Sources	Articles
*Physical Therapy*	14
*BMC Musculoskeletal Disorders*	8
*Cochrane Database of Systematic Reviews*	8
*Cureus Journal of Medical Science*	8
*Journal of Manipulative and Physiological Therapeutics*	6
*Manual Therapy*	6
*Osteoarthritis and Cartilage*	6
*BMJ Open*	5
*Journal of Back and Musculoskeletal Rehabilitation*	5
*Journal of Clinical Medicine*	5
*Musculoskeletal Care*	5
*Physiotherapy Theory and Practice*	5

**Table 7:** Top 10 most productive authors based on publication counts and fractionalized authorship.

Author	Articles	Articles Fractionalized
Buchbinder R	6	0.76
Arroyo-Morales M	5	0.61
Cantarero-Villanueva I	5	0.61
Hunter DJ	5	0.46
Boivin J	4	0.26
Boyle JA	4	0.26
Costello MF	4	0.26
Cote P	4	0.24
Fernandez-Lao C	4	0.49
Galiano-Castillo N	4	0.47

**Table 8:** Authors with the highest local citation counts in multimodal musculoskeletal rehabilitation research (1989–2026).

Author	Local Citations
Cedraschi C	16
Airaksinen O	14
Brox JI	14
Staal JB	14
Ursin H	14
Hildebrandt J	14
Klaber-Moffett J	14
Kovacs F	14
Mannion AF	14
Reis S	14

**Table 9:** Top nine globally highly cited guideline and clinical evidence papers in multimodal musculoskeletal rehabilitation.

Paper	Total Citations	TC per Year	Normalized TC
mcalindon te, (2014) OARSI guidelines for the non-surgical management of knee osteoarthritis	2153	165.62	16.59
zhang w, (2008) OARSI recommendations for the management of hip and knee osteoarthritis, Part II: OARSI evidence-based, expert consensus guidelines	2049	107.84	7.33
aıraksınen o, (2006) Chapter 4. European guidelines for the management of chronic nonspecific low back pain	1798	85.62	5.34
qaseem a, (2017) Noninvasive Treatments for Acute, Subacute, and Chronic Low Back Pain: A Clinical Practice Guideline from the American College of Physicians	1537	153.70	9.40
zhang w, (2007) OARSI recommendations for the management of hip and knee osteoarthritis, Part I: Critical appraisal of existing treatment guidelines and systematic review of current research evidence	553	27.65	4.00
van middelkoop m, (2011) A systematic review on the effectiveness of physical and rehabilitation interventions for chronic non-specific low back pain	516	32.25	7.40
hayden ja, (2021) Exercise therapy for chronic low back pain	397	66.17	12.13
ferreira ml, (2007) Comparison of general exercise, motor control exercise and spinal manipulative therapy for chronic low back pain: A randomized trial	312	15.60	2.26
sarzi-puttini p, (2005) Review for an American College of Physicians Clinical Practice Guideline	293	13.32	3.05

Abbreviations: TC, total citations; TC per Year, total citations per year; Normalized TC, normalized total citations; OARSI, Osteoarthritis Research Society International.

**Table 10:** Top 10 locally highly cited references shaping the intellectual core of multimodal musculoskeletal rehabilitation (1989–2026).

Summary	Paper DOI	Year	Local Citations	Global Citations	LC/GC Ratio (%)	Normalized Local Citations	Normalized Global Citations
Airaksinen O, 2006, Eur Spine J	10.1007/s00586-006-1072-1	2006	14	1798	0.78	5.44	5.34
Qaseem A, 2017, Ann Intern Med	10.7326/M16-2367	2017	12	1537	0.78	9.43	9.40
Ferreira ML, 2007, Pain	10.1016/j.pain.2006.12.008	2007	9	312	2.88	9.00	2.26
Van Middelkoop M, 2011, Eur Spine J	10.1007/s00586-010-1518-3	2011	9	516	1.74	10.64	7.40
Furlan AD, 2002, Spine	10.1097/00007632-200209010-00017	2002	5	128	3.91	3.00	2.10
Preyde M, 2000, Can Med Assoc J	N/A	2000	4	121	3.31	1.00	1.00
Poitras S, 2005, Phys Ther	10.1093/ptj/85.11.1168	2005	4	99	4.04	3.43	1.03
Medlicott MS, 2006, Phys Ther	10.1093/ptj/86.7.955	2006	4	195	2.05	1.56	0.58
Cantarero-Villanueva I, 2012, J Manip Physiol Ther	10.1016/j.jmpt.2012.10.007	2012	4	27	14.81	7.33	0.71
Van Doormaal MCM, 2020, Musculoskelet Care	10.1002/msc.1492	2020	4	120	3.33	16.57	6.67

Abbreviations: LC, local citations; GC, global citations; LC/GC Ratio, ratio of local citations to global citations; N/A, not available.

## Data Availability

No new data were created or analyzed in this study. Data sharing is not applicable to this article.

## Supplementary Materials

The following supporting information can be downloaded at https://www.mdpi.com/article/10.3390/healthcare14111564/s1. Figure S1: Time Slice 1 (1989–2005): Early unimodal biomedical rehabilitation themes. Figure S2: Time Slice 2 (2006–2015): Transitional exercise-based and guideline-oriented rehabilitation themes. Figure S3: Time Slice 3 (2016–2026): Contemporary multimodal and self-management-oriented rehabilitation themes. Figure S4: Top ten countries by publication volume and citation.

## Abbreviations

AAS	Altmetric Attention Score
AKW	Author Keywords
BC	Bibliographic Coupling
BMSK	Biopsychosocial Model in Musculoskeletal Rehabilitation
CBT	Cognitive–Behavioral Therapy
CR	Cited References
CTS	CiteSpace
GC	Global Citations
HTA	Health Technology Assessment
IASP	International Association for the Study of Pain
KWP	Keywords Plus
LC	Local Citations
LLR	Log-Likelihood Ratio
MCP	Multiple-Country Publication
MMR	Multimodal Musculoskeletal Rehabilitation
MSK	Musculoskeletal
NICE	National Institute for Health and Care Excellence
OA	Osteoarthritis
OARSI	Osteoarthritis Research Society International
PT	Physiotherapy/Physical Therapy
RCT	Randomized Controlled Trial
SCP	Single-Country Publication
SCIE	Science Citation Index Expanded
SM	Self-Management
TC	Total Citations
VOS	VOSviewer
WHO	World Health Organization
WoS	Web of Science
