# Supplementary figures and images for: Multimodal Musculoskeletal Rehabilitation in Clinical Practice: A Bibliometric and Altmetric Mapping Study (1989–2026)
Source: Healthcare (Basel). 2026 Jun 3;14(11):1564. doi: 10.3390/healthcare14111564 (PMC13256805; doi:10.3390/healthcare14111564)

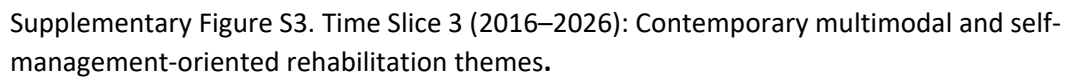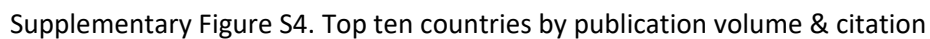

Supplement: Supplementary file 1 [file healthcare-14-01564-s001.zip › healthcare-4174886-supplementary.pdf]
